# Opportunities of Habitat Connectivity for Tiger (*Panthera tigris*) between Kanha and Pench National Parks in Madhya Pradesh, India

**DOI:** 10.1371/journal.pone.0039996

**Published:** 2012-07-16

**Authors:** Chinmaya S. Rathore, Yogesh Dubey, Anurag Shrivastava, Prasad Pathak, Vinayak Patil

**Affiliations:** 1 Indian Institute of Forest Management, Bhopal, India; 2 Madhya Pradesh Forest Department, Bhopal, India; Australian Wildlife Conservancy, Australia

## Abstract

The Tiger (*Panthera tigris*) population in India has undergone a sharp decline during the last few years. Of the number of factors attributed to this decline, habitat fragmentation has been the most worrisome. Wildlife corridors have long been a subject of discussion amongst wildlife biologists and conservationists with contrasting schools of thought arguing their merits and demerits. However, it is largely believed that wildlife corridors can help minimize genetic isolation, offset fragmentation problems, improve animal dispersal, restore ecological processes and reduce man animal conflict. This study attempted to evaluate the possibilities of identifying a suitable wildlife corridor between two very important wildlife areas of central India – the Kanha National Park and the Pench National Park – with tiger as the focal species. Geographic Information System (GIS) centric Least Cost Path modeling was used to identify likely routes for movement of tigers. Habitat suitability, perennial water bodies, road density, railway tracks, human settlement density and total forest edge were considered as key variables influencing tiger movement across the Kanha-Pench landscape. Each of these variables was weighted in terms of relative importance through an expert consultation process. Using different importance scenarios, three alternate corridor routes were generated of which one was identified as the most promising for tiger dispersal. Weak links – where cover and habitat conditions are currently sub-optimal – were flagged on the corridor route. Interventions aimed at augmenting the identified corridor route have been suggested using accepted wildlife corridor design principles. The involvement of local communities through initiatives such as ecotourism has been stressed as a crucial long term strategy for conservation of the Kanha-Pench wildlife corridor. The results of the study indicate that restoration of the identified wildlife corridors between the two protected areas is technically feasible.

## Introduction

The 21^st^ century has brought many conservation challenges to the fore. One very important and significant challenge that has evoked considerable scientific interest is the fragmentation of wildlife habitat. With rapidly expanding human populations and other competing land uses, areas that used to be continuous habitat have become broken and fragmented, isolating plant and animal populations contained within them. Habitat fragmentation is usually a time driven process that is innocuously initiated by human habitation or man induced habitat alteration and which eventually accelerates and results in complete isolation of once contiguous habitat. Populations thus isolated face survival pressures through increased competition for food and space and obligated risks in relation to disease outbreaks and episodic calamities such as fire and flood. Over a larger time span, species inhabiting isolated habitats also face the risk of extinction through mechanisms such as excessive inbreeding [1], [2].

The habitat fragmentation issue is of particular relevance to developing countries where most of the biodiversity rich tropical ecosystems are located. Natural ecosystems in many developing nations are currently facing an unprecedented threat from diverse competing pressures arising from a burgeoning human population and unregulated economic growth. India is one of the twelve mega-biodiversity nations of the world [3]. Of these, around 175 animal species are in the IUCN [4] red list threat category. India is also home to over 1 billion people many of whom live proximate to forest areas depending on them for their livelihood and subsistence. Urbanization, industrialization, infrastructure development projects, agriculture, grazing, deforestation, wildlife trade and poaching continue to create tremendous stress on pristine natural habitat and wildlife. As habitats shrink and populations become more isolated on ‘habitat islands’ studded in a matrix of alternate land use, serious questions on long term survival of many key species are now being asked. The status of large cats located at the apex of the food pyramid, is a grim reminder of the intense pressure that these animals face due to habitat loss. In the recent times, considerable scientific and media attention has been focused in India on large mammals – particularly large cats –and their conflict with man largely attributed to shrinking habitat. The fierce conflict between man and leopard in Mumbai [5] due to habitat loss; the very vulnerable and small population of 441 Asiatic lions *Panthera leo persica*) located in only 259 km^2^. of *core* forest habitat in Gir, Gujarat [6]; the highly vulnerable population of between 1706 tigers in India (as per the 2009–2010 census) threatened by habitat loss and poaching [7] are all indicative of the vulnerability that these fiercely territorial apex predators face today.

One solution to ameliorate the undesirable effects of habitat loss is to reverse the process of fragmentation by providing or rebuilding connectivity between isolated habitat patches through wildlife corridors. The idea of wildlife corridors was probably proposed for the first time by Wilson and Willis [8] as a means of conserving biodiversity based on the theory of island biogeography. A wildlife corridor has been defined as a “linear landscape element which serves as a linkage between historically connected habitat/natural areas, and is meant to facilitate movement between these natural areas” [9]. Creation of wildlife corridors has received much global attention during the last two decades. While the utility of wildlife corridors has been debated [10], [11], [12], it is largely believed that wildlife corridors facilitate animal dispersal from isolated habitats and help counter biological processes that lead to species extinction [13], [14], [15], [16], [17], [18], [19], [20], [21], [22], [23]. While the idea of connecting fragmented patches may appear simplistic at first sight, the identification, design and development of wildlife corridors in large landscapes presents unique challenges [24]. Beier & Loe [25] observe that the critical features of a wildlife corridor are not its physical traits such as its length or width or vegetation but rather how well a particular piece of land fulfills several functions like survival of species, facilitation of travel, migration, mate finding of wide ranging animals, propagation of plants, genetic interchange, movement of populations in response to environmental changes and natural disasters and re-colonization of habitat areas by individuals. The importance of wildlife corridors for tiger conservation in India has also been significantly reiterated by Jhala,Qureshi,Gopal and Sinha [7].

The present study was undertaken to explore the possibilities of establishing connectivity between two very important wildlife areas – the Kanha national park and the Pench national park – in the central Indian state of Madhya Pradesh.

## Methods

### Study Area

The Kanha National Park – which is located in the Mandla, Balaghat and Dindori districts of Madhya Pradesh (MP) – covers an area of around 940 km^2^. An additional area of about 1000 km^2^ constitutes the buffer zone for Kanha. Kanha was one of the first nine protected areas to be brought under the ambit of the Project Tiger launched by the Government of India in 1973. Kanha is one of the richest biodiversity areas in India with around 22 species of mammals and 300 species of birds. The 2010 tiger census of India showed Kanha to have population of 60 tigers [7]. The Pench National Park and tiger reserve are spread over the Seoni and Chhindwara districts of Madhya Pradesh. The core area of the Pench National Park in MP is around 293 km^2^ while the buffer area of the Pench tiger reserve is around 758 km^2^. The Pench National Park is also very rich in biodiversity with around 20 species of mammals and around 300 species of birds. As per the 2010 tiger census of India, the Pench National Park in MP had 54 tigers [7]. Both Kanha and Pench National Parks are administered by the Madhya Pradesh Forest Department. Interestingly, the Pench National park contiguously extends into the state of Maharashtra covering an area of 257 km^2^. in that state and is administered there by the Maharashtra Forest Department. The Maharashtra Pench area has 11 tigers as per the 2010 tiger census [7].

The study opted to concentrate on one primary focal species – the Tiger (Panthera *tigris*) for delineation of suitable corridor paths between Kanha and Pench National Parks. There were some important factors to consider only one focal species for this study. In terms of conservation focus, the tiger is the most important species and the central Indian forests hold the largest single population of tigers. Currently, a very high priority is being accorded to tiger conservation in India due to their declining numbers and shrinking habitat. While no detailed studies are available, some earlier work [25] seems to indicate that sympatric carnivores in these forests – particularly wild dogs – would in all likelihood also, use corridors designed for tigers due to an overlap of prey species.

Kanha and Pench national parks are separated by a road distance of approximately 200 km and a large part of the intervening area between these two national parks is covered by forests which are under the control of the Madhya Pradesh Forest Department (MPFD). These intervening forests between Kanha and Pench are territorial forest divisions managed conventionally as prescribed by forest working plans. The continuity for forests in these areas is unbroken for large stretches even though at some places discontinuity and fragmentation in forest cover can be observed. These intervening stretches of forests offer promising opportunities for developing a corridor area suitable for the movement of Tiger between these two very important protected areas. Currently however, the forest area between Kanha and Pench is not viewed as a wildlife corridor and is not managed primarily for wildlife values. This study specifically focused on the use of Geographic Information System (GIS) modeling to identify optimal corridors for movement of tigers using Least Cost Path (LCP) analysis.

### Satellite Image Analysis

Digital image analysis of remotely sensed satellite data was done on the ERDAS Imagine 8.6 digital image analysis system to determine forest cover status for habitat mapping. The study area was covered by four Indian Remote Sensing Satellite -1D LISS III scenes. Data for the month of November 2002 was chosen keeping in mind defoliation of teak beyond this time. As monsoon activity concludes in central India around September end, cloud free data for the study area was available for the month of November. Radiometric correction was applied to the LISS data. Satellite images of the study area were geo-referenced to 1∶250,000 topographic map sheets of the Survey of India. The transverse mercator projection was used with WGS84 spheroid & datum and other projection parameters as used by the Survey of India for its digital topographic map database. All four satellite images were geometrically rectified using appropriate GCPs selected from the topographic map sheets. The geometrically corrected raster images were mosaicked to prepare a single dataset covering the entire study area using standard mosaic routines available in ERDAS and color differences at the edges of adjoining images were removed using the histogram matching technique. The image was classified into six vegetation classes (Table 1).Post classification smoothing was done using a 5×5 median statistical filter to merge stray isolated pixels. Ground verification of the vegetation classification was done traversing the landscape area for various classes using the Leica GS5 GPS receiver system and Garmin 12 XL GPS receiver.

**Table 1:** Vegetation classes and description.

Class ID	Class Name	Class Description
1	Uniform Teak forest/plantation	Mostly pure teak stands
2	Teak mixed forest	Teak and other species are found in about equal proportions.
3	Teak bamboo forest	Teak forest with bamboo under story
4	Mixed or Miscellaneous forest	Miscellaneous forest with no dominant species
5	Mixed Bamboo forest	Mixed forest with bamboo under story
6	Bamboo Mixed forest	Mixed forest but with high density of bamboo

### GIS

LCP analysis was used to determine optimal corridor paths between the two national parks. LCP is a multi-step process performed on raster surfaces in a GIS environment. Considering the area between the points between which connectivity is being explored, it computes a composite ‘cost of movement’ score for every cell in the intervening landscape grid by considering factors that would promote or impede movement of the focal species. The higher the calculated movement cost for a grid cell, the less is the likelihood that the animal will move there. Using this data, the GIS cost path function predicts the most likely route to be taken by the target species by connecting cells that have the least cost of movement. The focal species based Least Cost Path analysis used in the present study has been used in many similar studies for delineating and designing wildlife corridors [26], [27], [28], [29], [30], [31].

In a departure from the focal species based corridor development approach however, Beier & Brost [32] have recently suggested using land facets, which are recurring areas of uniform topography and soil characteristics, as a more stable basis for identifying wildlife corridors and for conservation planning. While the land facet based corridor identification might also use least cost modeling, it is divergent from the focal species oriented approach in that it does not rely on current land cover maps but rather on the more stable topographic and soil attribute parameters which in conjugation with climate, fundamentally determine the composition of species assemblages at a given site. The land facet approach argues that given the developing climate change scenario, the assemblage of species might change with changes in climate and as a consequence, corridors based on transient variables such as land cover might become unviable in the future. However, linkages based on connectivity of land facets will be more durable as they are derived from fundamental rather than transient attributes. The land facet based corridor approach is however currently evolving and might be at present constrained for data particularly better soil maps limiting somewhat, accurate delineation of land facets [32]. It has also been suggested by Beier & Brost [32] that linkages developed using the land facet approach might result in inclusion of half or more areas of the landscape as part of the corridor creating ambitious conservation goals. Majka [33] after testing the land facet approach in three Arizona landscapes cautions that the land facet approach be used to complement and not replace corridors delineated using focal species based procedures.

All GIS work was done on ArcView GIS 8.3. Base maps pertaining to drainage, contours, road network, rail network, and water bodies at 1∶250000 scale were procured in digital form from the Survey of India – the national mapping agency for India. Forest compartment maps were procured as paper copies from the Madhya Pradesh Forest Department and integrated in the geographic database after digitization.

### Cost Path Model Development

For the present study, six key variables were considered in defining movement preferences of tigers (Table 2).

**Table 2:** Variables and description.

Variable	Model Context
Habitat	Assessment of suitable habitat with reference status of prey base for Tiger
Perennial Water bodies	Location of perennial sources of water in the corridor landscape
Road Density	Status of road network in the corridor landscape which may act as a movement barrier
Railway Track	Status of railway network in the corridor landscape which may act as a movement barrier
Settlement Density	Status of permanent human presence in the corridor landscape
Total Forest Edge	Assessment of forest fragmentation and status of covered or uncovered areas

The choice of variables was based on published literature sources on tiger ecology & migration [34], [35], expert consultation and similar studies on cougars [18], [22], [30], [31].

#### Habiatat (H)

The habitat layer ranks zones preferred by prey species of tiger in the study area. Assessment of preference is based on field data of pellet counts associated with each cover type. Habitat zones where prey populations are abundant are likely to be preferred by the tiger in comparison to those where they are scarce or absent. Consequently, cost of movement to high prey abundance areas will therefore be less. Habitat value rankings (Table 3) were assigned to each cover type through a delphi ranking process keeping in view prey abundance data. As can be seen, areas dominated by bamboo are preferred by tiger prey species as sufficient browse is available at approachable height.

**Table 3:** Habitat types and cost rank.

S. No	Habitat Type	Cost
1	Teak	4
2	Miscellaneous	3
3	Teak Mixed	2
4	Bamboo Mixed	1
5	Mixed Bamboo	2
6	Non-Forest	10

#### Perennial water bodies (PWB)

Water availability is a critical requirement. In the study area landscape, water becomes particularly scarce during the summer months. At such times, prey populations also converge at water sources providing predators with hunting opportunities. Movement into areas proximate to perennial water sources would therefore be favored. The water grid was constructed by combining the perennial streams from the river coverage and lakes from the classified satellite map. The distance from these perennial sources of water was represented as a water distance grid and was calculated using the straight line function in the Spatial Analyst module in ArcMap. The straight line function calculates the Euclidean distance for each cell from the nearest source (water) cell. The range of distances obtained was reclassified into nine categories with corresponding cost rank values (Table 4).

**Table 4:** Distance from water bodies and cost values.

S. No.	Type	Cost
1	0–1 km	0
2	1–2 km	1
3	2–3 km	2
4	3–4 km	3
5	4–5 km	4
6	5–6 km	5
7	6–7 km	6
8	7 & above	7
9	Actual Water Bodies	10

The area occupied by water bodies were treated as having a high movement cost as they might act as movement barriers.

#### Road density (RD)

Roads act as a barrier to movement. An area having a high density of road would be avoided as compared to an area with few or no roads. Apart from other things like road dividers, interstate roads which usually have a very high traffic volume, further amplify the barrier effect due to constant noise and vehicular movement. A road density grid was created using the road coverage along with data on Passenger Car Unit (PCU) acquired from the Madhya Pradesh highway department. PCU data was attached to each road segment and road density with respect PCU was calculated using the LINEDENSITY function. The entire range of the resultant values was divided into High, Medium, Low and Absent categories. Areas where road density is high present a higher traversing cost as compared to areas having low road density. Cost rank values for road density are presented in Table 5.

**Table 5:** Road density and cost rank.

S. No	Category	Range (pcu/m)	Cost
1	High	6 to 11	10
2	Medium	4 to 6	5
3	Low	0.1 to 4	2
4	Absent	0	0

#### Railway (R)

The study area landscape is also bisected by railway lines at a few places. While railway tracks are not formidable physical barriers, noise and vibration emitted due to passing trains can act as deterrents to movement. For the purposes of this study therefore, areas where a railway line is present was considered as a movement resistance zone for the tiger. Cost ranks assigned by presence or absence for this variable are presented in Table 6.

**Table 6:** Railway track and cost rank.

S. No.	Category	Cost
1	Presence of railway line	10
2	Absence of railway line	0

#### Settlements (S)

Areas of human settlements are usually avoided by migrating animals. The denser and more populous an area, the more formidable it is as a movement barrier. Tigers, under normal circumstances, avoid traversing through such areas. To derive a human settlement grid, census data for each settlement was attached to settlements in the study area. POINTDENSITY function was used to calculate population density. The resultant density range was used to categorize settlements. Cost rankings for this variable are presented in Table 7.

**Table 7:** Human settlement cost rank.

S. No.	Category	Range (pop/m^2^)	Cost
1	High	0.0015 – 0.0022	10
2	Moderate	0.001 – 0.0015	6
3	Low	0 – 0.001	3
4	Absent	0	0

#### Total edge (TE)

Tigers are solitary and elusive animals. Fragmented forests present expanses of open spaces to the ranging animal to negotiate which it might possibly avoid. In landscape ecology terms, such fragmented habitats have a high proportion of forest edge as compared to core areas where forest cover is unbroken [36], [37]. Edge is defined as the portion of an ecosystem bordering its perimeter, where influences of the adjacent patches can cause an environmental difference between the interior of the patch and its edge. This edge effect includes a distinctive species composition or abundance in the outer part of the landscape patch [38]. Species like the tiger, which prefer core habitats, generally avoid areas with high edge density. Estimation of edge per unit area in the study area was thus of importance in terms of inhibiting or promoting movement of the focal species.

Total Edge (TE) – a landscape level metric – has been defined as the sum of the lengths (m) of all edge segments in the landscape. If a landscape border is present, TE includes landscape boundary segments representing ‘true’ edge only (i.e. abutting patches of different classes). If a landscape border is absent, TE includes a user-specified proportion of the landscape boundary. Regardless of whether a landscape border is present or not, TE includes a user-specified proportion of internal background edge. Total edge is an absolute measure of total edge length of a particular patch type [39].

Total Edge in a landscape is given as:

TE  =  E

Where E  =  total length (m) of edge in landscape.

TE ≥0, without limit

TE  = 0 when there is no edge in the landscape; that is, when the entire landscape and landscape border, if present, consists of a single patch and the user specifies that none of the landscape boundary and background edge be treated as edge.

As TE is a landscape level metric, if it were computed for the study area landscape it would result in a single value indicating total edge length in meters for the whole landscape. The requirement for the present study was to calculate total edge in smaller unit areas covering the entire study landscape. In order to achieve this, the study area was first reclassified into forest and non-forest areas by merging all forest classes into one and then tessellated using hexagon units. Each hexagon unit was treated as a landscape and TE calculated for it. In this manner it was possible to compute TE for contiguous regions covering the entire landscape.

The total edge landscape index was calculated using Patch Analyst which employs Fragstats in the background for metric calculations [39]. The total edge length between different land use classes was calculated within each hexagon. Cost rank values obtained by categorizing total edge are summarized in Table 8.

**Table 8:** Forest edge estimate and cost rank.

S. No.	Category (Meters)	Cost
1	100–27,000	0
2	27,000–35,000	4
3	35,000–59,000	6
4	59,000–77,000	8
5	77,000 & above	10

**Figure 1:**
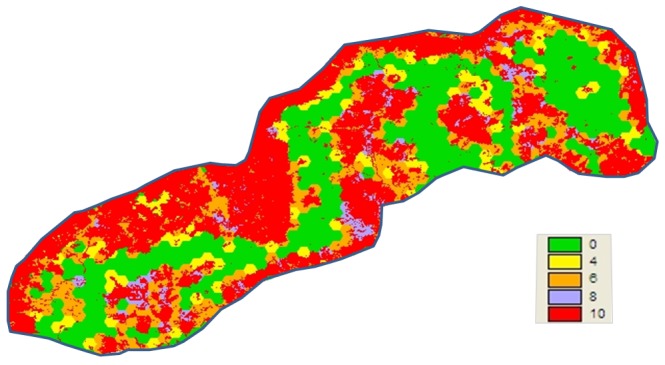
Edge cost grid derived from categorization of computed edge lengths.

Figure 1 shows the categorized edge cost grid. Areas in green show zones where cost of travel is minimal as they have very little edge areas.

Integrating all parameters together, it was possible to arrive at a unified equation to calculate cost of Movement for the tiger in the landscape. The Composite cost of Movement (CM) is defined as:
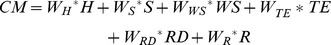



Where,

CM = Composite Movement Cost

H = Habitat Cost

S = Settlement Cost

WS = Water Source Cost

TE = Total Edge Cost

RD = Road Density Cost

R = Railway Cost

WH = Importance weight for Habitat

WS = Importance weight for Settlement

W_WS_ = Importance weight for Water Sources

W_TE_ = Importance weight for Total Edge

W_RD_ = Importance weight for Road Density

WR = Importance weight for Railway

and



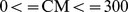



Expert opinion has been commonly used in conservation planning, habitat suitability and corridor selection [40], [41], [42], [43], [44], [45]. Justifying the use of expert opinion in corridor design, Beier,Majka and Jenness [46] have highlighted that while such models may be subject to errors and uncertainties, they are easy to create, do not require detailed field data and can be applied to multiple study areas. There has been, however, some criticism on the use of expert opinion in assignment of costs to landscape features used in LCP models on account of a general lack of validation of using empirical data or lack of assessment of model sensitivity to errors in cost assignment [47]. The present study relied on expert judgment to weight cost surfaces and inform LCP analysis for identification of suitable corridor path as in the given data availability scenario, expert judgment constituted the most informed mechanism to factor in tiger movement preferences.

For calculating weightage, five leading experts on tiger behavior were contacted with a questionnaire to rate various model parameters on a scale of 1 to 5 for their importance with respect to tiger movement. An additional parameter of slope was also introduced in the questionnaire to get an expert perception if slope could be a major impediment in movement of tigers across a landscape (Table 9).

**Table 9:** Weights to different model variables by experts (E1–E5).

	Weightage Assignment					
Parameters ↓	E1	E2	E3	E4	E5	Average
Cover	2	5	5	4	4	4
Food availability/Prey base	2	4	3	3	5	3.4
Water availability	2	2	4	1	4	2.6
Presence of roads	1	1	0	1	3	1.2
Human habitation	1	3	2	4	5	3
Presence of railway track	1	0	0	1	2	0.8
Slope	0	0	0	0	1	0.2

As can be seen from Table 9, cover, prey base, water availability and human habitation (avoiding inhabited areas) rank high on a majority of responses. All experts indicated that slope probably would not be a factor impeding tiger movement. Likewise presence of roads and railway tracks also ranked low as impeding factors. Trial runs were performed using various weighting schemes to better assess their impact on predicted migration routes. Considering various possibilities, it was observed that the following three run options adequately captured the variability introduced by weights given by experts in the cost path calculations. The generation of the following options also helped to better understand model sensitivity to weights assigned for cost surfaces.

#### Run option 1 (RO1)

Run Option 1 was evolved considering all cost themes to be equally important. The cost of movement with this weighting scheme was defined as follows:
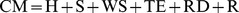



#### Run option 2 (RO2)

Run option 2 was based on the E2 weighting scheme (Table 9). In this weighting scheme, the presence of good forest cover with a weight of 5 was the most important requisite for movement of the dispersing animal. Suitable habitat with a good prey base was next in order of relative importance with a weight of 4. Human habitation, human population density, distance to water and road density were assigned weights of 3, 2 and 1 respectively. The cost of movement with this weighting scheme was defined as follows:
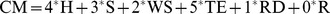



#### Run option 3 (RO3)

RO3 was evolved based on the E1 weighting scheme (Table 9). In this weighting scheme, habitat type, edge, and distance from water were presumed to be twice as important as the other contributing factors. Thus, habitat, total edge, and distance to water cost layers were multiplied by a weight factor of 2 while the other variables did not get any weights. The cost of movement as per this weighting scheme was defined as follows:
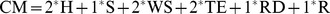



### Prey Abundance

Sidensticker [48] notes that if human induced mortality is discounted, the distribution and density of prey populations rather than vegetation parameters determine tiger density. Cost estimations for suitable habitat for movement of tiger were accordingly based on correlating prey abundance with cover type. These estimates however could not be validated with actual tiger presence data through direct sighting or tiger scat data as the proposed corridor area did not have a significant tiger population and tiger scat was very infrequently encountered for it to be used to make reliable inferences in this context.

In order to evolve cost rankings for habitat type (Table 3), field studies were undertaken to correlate prey abundance with cover type. A number of direct and indirect methods of estimating mammal densities have been used in tropical forest [49], [50], [51], [52], [53], [54], [55], [56], [57], [58]. Estimates based on indirect methods usually involve counting animal droppings, whereas direct methods use visual sightings. For the present study, pellet/dung (henceforth called only pellet) counts were undertaken to estimate an index of relative abundance of ungulates in the study area. The broad sampling strategy involved visiting patches of different habitat types and collecting pellet data in belt transects. Care was taken to sample each habitat type in approximately the same proportion as it occurs in the study area.

Each belt transect or plot was of size 10m x 2m. A transect was laid in each sampled patch and on each transect 5 plots of the mentioned size were placed at 100m from each other. Sampling was carried out by a team of 2–3 persons including one researcher and at least one local person. The researcher was trained in identification of pellet groups during earlier visits to the forests in study area. In each plot, this team carefully searched for pellets of forest ungulates found in the study area. Pellet groups were located and identified to the species and were recorded in pre-designed datasheets. A pellet group was identified as a group of pellets numbering more than 5 and a result of one event of defecation of an individual animal. In case of Nilgai and Chowshinga which create large latrines by defecating at the same spot, a latrine was counted as one pellet group.

The field surveys were undertaken during December 2004 to March 2005. The strategy was to collect samples from the whole stretch of the landscape. At the same time, it was kept in mind to collect samples from each habitat category roughly in its proportion to the whole forest area. Sites were selected using land-use map of the study area which depicted these habitat categories. Each site was carefully selected so as to avoid edges of habitat patches. At each of these sites, a transect of 0.5 km length was walked. At every 100 m along this transect pellet plots were laid. A total of 79 transects were walked and the total number of plots studied was 395.

Tiger’s preferred prey includes large forest ungulates [59]. In the study area, this group is commonly represented by chital (*Axis axis*), sambar (*Cervus unicolor*), wild-boar (*Sus scrofa*), and bison (*Bos gaurus*). Others like barking deer (*Muntiacus muntjak*), chowshinga (*Tetracerus quadricornis*) and nilgai (*Boselaphus tragocamelus*) were also rarely encountered. Within the forest habitat, it was assumed that ungulates show preferences for different habitat conditions imposed by the structure and composition of forest. Thus an index of ungulates’ use for each forest type was estimated.

### Prioritizing Routes

After deriving a likely route of travel for the tiger, additional information was required to adequately design a dispersal corridor. Key questions like the width of the proposed corridor or management prescriptions for the habitat surrounding the corridor route needed to be evolved in order to suitably develop and implement the corridor across the landscape. With reference to width of the corridor, some workers have suggested that the width of the corridor should ideally be determined using home range data. For the focal species to permanently occupy the corridor, it should be as wide as one home range of the dispersing animal [22], [60]. Paucity of sufficient habitat and large home ranges of tigers, however, completely discounted the possibility of a one home range wide corridor in the study area. Tigers have very large home ranges which have been reported to be up to 30–40 kilometers or even larger.

In more practical terms, broad guidelines with reference to width and habitat aspects while designing wildlife corridors have been suggested by some workers. Bond [61] suggested that the corridor should be as wide as possible. The corridor width may vary with habitat type or target species, but a rule of thumb is about a minimum of 1,000 feet wide but larger if possible.

Maximization of land uses adjacent to the corridor that reduce human impacts are also desirable [24]. Also, isolation effects along corridors can be offset by having surrounding habitat similar to that found within corridors [62].

## Results

### Habitat Mapping

The pellet count data was analyzed for estimating mean pellet density for each habitat type. This pellet density was first estimated as number of pellet groups per plot. Table S1 shows these densities with an estimate of Standard Error (SE) of each. These estimates were then converted to number of pellet groups per Hectare (Table S2).

Based on these pellet densities, the habitat types were ranked to know the preferences of different ungulate species. For each habitat type, ranks for all ungulate species were summed up to arrive at a cumulative ranking of all prey bases for each habitat type. For all species of prey and livestock, the habitats are ranked based on pellet density estimates. The habitat with highest pellet density is assigned rank 1 and the lowest, including 0, a rank of 6 (Table S3). While there are great, almost antagonistic differences among various prey species in their habitat selection e.g. Sambar and Chital, a summation of all ranks gives a cumulative rank to each habitat type. This can be used as an indicator of the prey base rank of each habitat. According to these rankings, Bamboo Miscellaneous is the most preferred habitat and Teak the least preferred.

Table S4 gives an account of zero counts i.e. the number and proportion of plots in each habitat type which recorded absence of pellet groups of a particular species. The percentage of zero count plots to all 395 plots in the bottom row indicates the abundance of prey species in relation to each other over the entire study area. It was found that Chital pellets were absent in less number of plots (79%) as compared with others which meant that Chital was found to be the most abundant animal in the study area. In contrast, barking deer pellets were not found in 98% of the plots indicating that barking deer was the least abundant of the studied animal species.

### Corridor Delineation

Three starting cells (from cells) were identified at the periphery of the Kanha National Park and a destination cell (source cell) was identified at the periphery of the Pench National Park end of the landscape. The three run options as described earlier were computed using cost path functions.

**Figure 2:**
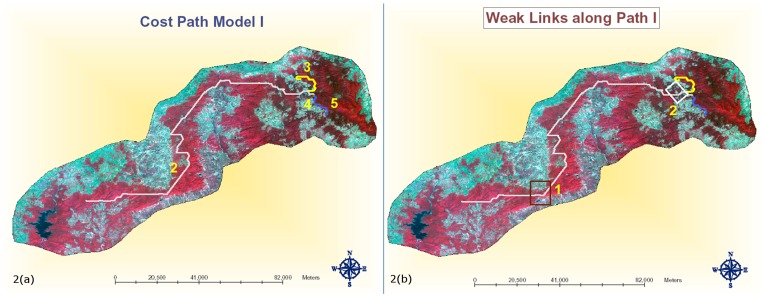
a. Optimal tiger migration path based on run option 1 weight scheme being equal. b. Weak links along the path indicated by rectangles.

**Figure 3:**
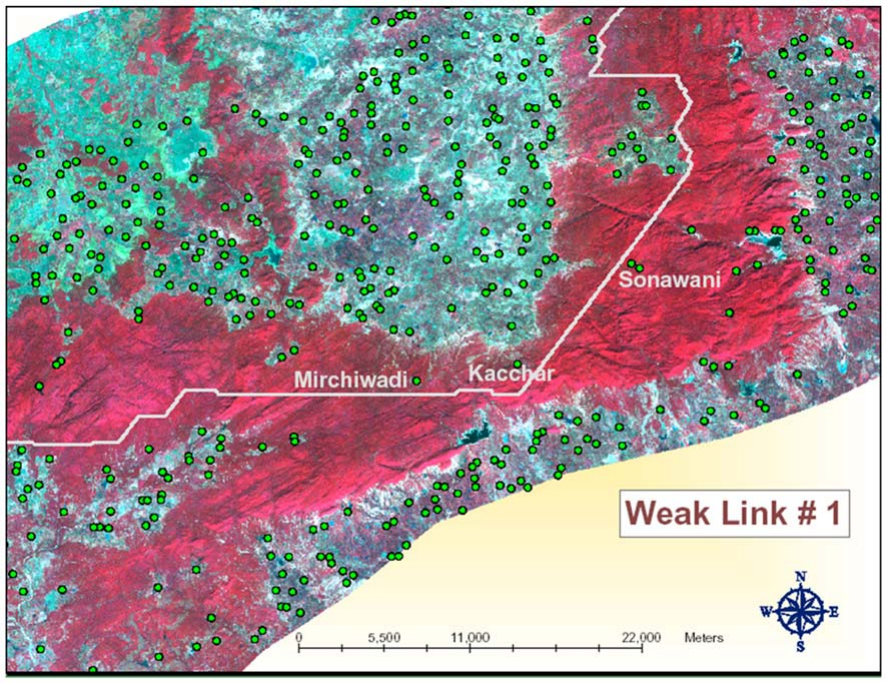
Weak link 1 identified by Run Option 1.

The first run option – RO1– resulted in the optimal path shown in Figure 2*a*. Only one major path could be computed across the landscape using RO1 parameters. The path starts from the top edge of the Kanha National Park and largely runs through forested areas reaching the source cell at the periphery of the Pench national park. Table S5 highlights length of various segments of this optimal patch. Overall, the shortest connecting link from Kanha to Pench from this option is around 171 kilometers (segment 2+4) across the landscape. The RO1 corridor path has two weak links (Figure 2*b*) where connectivity is significantly jeopardized. Weak links in a corridor path can be described as those very fragile areas along the predicted corridor path that are highly constricted in width, have high forest fragmentation or have a high degree of anthropogenic presence and pressure. The first of these weak links, annotated as 1 in Figure 2*b* *,* has been found to be just 4 kilometers wide (Figure 3). The area is completely surrounded by agriculture fields. Water sources in this area are very scarce. The Nahle Sarra water tank is the major potential water source for migrating animals being situated outside forest area poses considerable risk for the migrating animal. The major villages inside the forest in this weak link are Mirchiwadi, Kacchar, and Sonawani. Even though human population of these villages is not more than 50, the livestock population is considerable creating grazing pressures in this area. The major vegetation type that forms the core part of this weak link area is Bamboo Miscellaneous. The Second weak link along the RO1 path annotated as 2 in Figure 2b is composed of a very narrow strip of vegetation. Human settlements and agrarian practices around this area are widespread and have considerably fragmented the forest. Surpati and Jhangul are the major human settlements located near this weak link. The human population of each of these villages is around 200. The Banjar river flowing through this areas is said to remain largely dry for most parts of the year except the rainy season. This area is characterized by miscellaneous vegetation and is highly fragmented with forest land interspersed with agricultural area.

**Figure 4:**
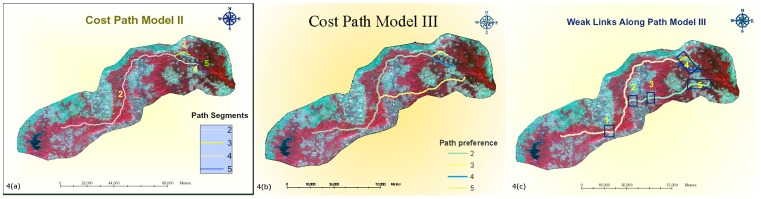
a. Cost Path identified by Run Option II. b. Cost Path identified by Run Option III. c. Weak links along path identified by Run Option III.

The second run option – RO2– resulted in the optimal paths shown in Figure 4*a*. Two paths emerge with this run option. One of these paths is segment 3 while the other path is segment 4 both of which merge into common path 2, just as they leave the Kanha tiger reserve area. The common segment 2, which traverses through the major part of the corridor landscape apparently looks quite similar to the common segment (2) generated by RO1. Careful observation however reveals that there are important differences in the RO segment 2 which has moved eastward in some portions of the landscape when compared to the common segment of RO1 (Figure 3). The shortest connecting link from Kanha to Pench resulting from this option is around 161 kilometers (segment 2+4) which is about 10 kilometers shorter than route predicted by RO1. Like RO1, the predicted migratory path has two weak link areas. The area where weak link 1 is located in the RO2 path is the same as in RO1. However, weak Link 2 in the RO2 path (the area near west boundary of Kanha Tiger Reserve) appears to be extended due to segment 3 path in RO2. The forest area is severely fragmented with interspersed agricultural land. Many small villages are located in this area of which the major villages include Kamta, Tatri, and Jhangul. The human population and livestock population in the area is substantial. The habitat type is largely miscellaneous with scanty Bamboo miscellaneous vegetation near Jhangul. In some areas, teak (old plantation) is also present.

The third run option – RO3– resulted in the optimal paths shown in Figure 4*b*. This option was based on the weighting scheme in which cover, prey and availability of water were considered twice as important as other model variables. RO3 results in three path options. While the common segment and path option 3 and 4 are quite identical to RO2, segment 5 starting from the lower periphery of the Kanha Tiger reserve, traverses a course bordering the southern periphery of the landscape before merging midway with the common segment 2 at Lalbarra. Segment 2+3 in this run option has a total length of 151 kilometers, segment 2+4 has a total length of 158 kilometers while segment 2+5 (from the point where 5 merges with 2) totals around 145 kilometers. It is worth mentioning that the length of segment 2 in the RO3 option is 33 kilometers shorter than that in RO1 and 11 kilometers shorter than RO2. This is because in RO3– unlike in RO1 and RO2– segment 2 terminates at the point where it is met by segment 3.

Figure 4*c* shows weak links along RO3 corridor path. While weak links 1, and 4 are the same as those mentioned in the RO1 and RO2 paths,two new weak links annotated as 2 and 3 (Figure 4*c*) appear in the RO3 path. These weak links suffer from severe fragmentation of forest due to anthropogenic pressures and cultivation. The areas have considerable human presence in surrounding proximity. Prominent villages include Durenda and Tengni Khurd. The Wainganga river flows vertically down to bisect the forest patch.

Considering the predicted path generated by RO3 as a more likely route, we proposed corridor development strategies based on a broader area proximate to the predicted corridor path. Accordingly, in a bid to maximize corridor width, a 2–6 kilometer buffer was demarcated around the predicted corridor route. One of the six segments of the predicted corridor path was overlaid on top of the satellite image. Such an overlay provides a good idea of the status of forest fragmentation in various buffer zones and the difficulty in having a uniformly wide corridor width. The buffers were also overlaid on top of compartment boundaries to identify those compartments that fall around the corridor and to understand distribution of forest cover within them. This resulted in identification of weak links that can be taken up for development of corridor. Bhamni range near Jhangul was one such prominent weak link identified with respect to long term corridor sustainability. In this forest range, to assess the ground situation in terms of wildlife-human interactions, wildlife status, and forest cover quality nine villages were surveyed using a questionnaire. Data gathered revealed that local people had strong emotional and cultural ties with the surrounding forest areas and acknowledged the role of the forest in terms of their livelihood and sustainability of wildlife. They are actively involved in protecting local forests via “Van Suraksha Samiti” (Forest protection committee). However, the younger generation did not appear to be entirely enthusiastic about the importance of conserving tiger habitat given the backdrop of pressing concerns relating to livelihood issues and the potential for man-animal conflict.

Based on the buffer overlay on top of the satellite image and the ground survey, it was observed that most of the corridor area possesses sufficient canopy cover. We also found the need of understory development and availability of open grasslands as important factors in maintaining prey abundance throughout the proposed corridor. Bamboo vegetation was observed to be in its recovery stage at several places; hence, we also recommend special attention towards protection and regeneration of Bamboo as understory vegetation for herbivores. Ground hugging forest fires evidenced at many places throughout the weak link areas like Jhangul range resulted in destruction of the understory vegetation. Control measures for forest fires are therefore necessary for long term corridor sustainability.

Availability of water was identified as a key issue in enhancing the feasibility of the proposed wildlife corridor as most of the water sources go dry during summers. We analyzed the distances between major perennial water bodies along all the segments of the corridor and found the distance to range between 12–29 km. To create an optimal water availability scenario for the area, water should be present within 5 km from any point of the corridor. We identified several forest compartments for creation of artificial water holes to fill in the gaps (Table S6).

**Figure 5:**
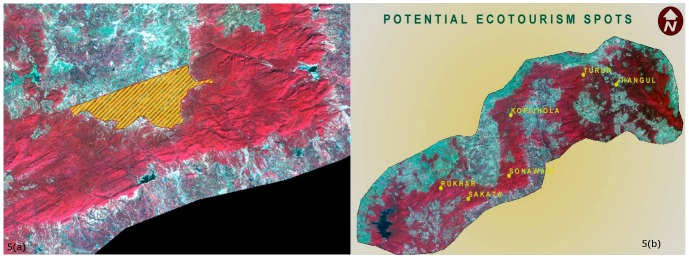
a. Ari weak link showing required land parcel on the satellite image. b. Potential ecotourism spots across the corridor.

Looking at the long term corridor establishment and conservation process, we also identified a few land parcels which are currently under private ownership, for acquisition and assimilation into the corridor area. This was especially required for areas surrounding the weak links to ensure connectivity. One such area was Ari weak link depicted in Figure 5*a*, where the probable land parcel for acquisition is displayed using a hatched polygon. We are of the view that if acquisition of such land for corridor was possible then it could be restored to its original vegetation composition in future. Using topographic maps for the area we identified the settlements for Lalburra-South Lamta weak link as well as East Baihar and Bahmni weak links, which can be considered for possible land acquisition. Given the fact that land acquisition has become a politically nuanced subject in India, this can be a challenging task in which case alternate links in the corridor as brought out by this study and which are under the control of the state forest department would have to be appropriately consolidated.

Identifying and promoting locations suitable for ecotourism was one of our key recommendations. Ecotourism has been already adopted in several regions of India attracting a growing number of tourists and possibly raising funding for the conservation [63]. The Kanha and Pench National Parks area is world famous for being action theatre of the Jungle Book by Rudyard Kipling. This study identified a few sites across the corridor landscape based on their potential for scenic beauty, animal sighting, proximity to local communities, access, residential infrastructure and geographical spread which could possibly act as ecotourism hubs where local communities could be involved. In particular, Rukhar, Sakata, Sonawani, Kopijhopla, Turur, and Jhangul (Figure 5*b*) hold considerable potential for initiating an integrated ecotourism programme. While there is a tremendous ecotourism potential in the area, its implementation might be quite challenging in terms of defining clear set of operating guidelines identifying mechanisms of equitable sharing of revenues with local communities [64].

## Discussion

Considering all options described above, it was observed that corridor paths emerging from RO3 provide more diverse connectivity options when compared to RO1 and RO2, if a holistic long term perspective is to be taken in terms of corridor development in the region. One of the primary reasons for the superiority of RO3 over RO1 and RO2 is the alternate connectivity route provided by link segment 5. This new connecting path passing through Lalbarra, South Lamta, North Lamta, West Baihar, East Baihar and tapering into the Kanha National Park has not been delineated in RO1 and RO2. The availability of segment 5– even though seemingly difficult due the highlighted weak links – considerably expands connectivity options in the whole landscape as forests in the Balaghat forest division (south of Kanha) also conjoin segment 5 forests. This opens up new avenues of animal dispersal from even other areas down south not specifically considered by this study. This connectivity is clearly visible on satellite imagery of the larger landscape. Hence the route delineated as a result of RO3 was considered the most suitable corridor option for focusing on development of a corridor even though RO1 and RO2 are valid movement corridors which might also be potentially utilized by dispersing tigers. As the forest land for purposes of effective management is divided into administrative units like circle, division, range and compartments, it was felt important that the corridor path generated by RO3 be overlaid on forest compartments falling in various forest ranges to provide a administratively familiar ground reference for the Madhya Pradesh Forest Department field staff developing and managing the corridor. Table S7 lists forest ranges and compartments through with the RO3 corridor passes with the exception of the Bahmani range for which compartment maps were not available at the time of this study.

The present study covered a very large landscape focusing primarily on finding potential corridor alternatives connecting two major protected areas in central India. While the study isolated and recommended a preferred corridor path between the two bearing high potential for development, the study faced limitations due to paucity of detailed field data at this scale on tiger sightings and current use of the proposed corridor habitat by the target species. The forest department, till the period in which this study was conducted, did not keep detailed systematic scientific records on habitat use, prey abundance, predator abundance, target species movement and gap areas all of which could have further informed our study.

The effectiveness of the LCP method in delineation of wildlife corridors has been questioned on the grounds of over reliance on remotely sensed habitat maps, use of expert opinion in assigning costs and ambiguity on deciding on the length and width requirements of the proposed corridors cautioning that LCP based corridor studies need to justify the above in terms of biological or empirical foundations [46]. While the criticisms and cautions in use of the LCP method are noteworthy, there have been many studies that have used the LCP method quite effectively in identification of wildlife corridors and predicting animal dispersal [45], [65], [66], [67], [68]. The present study tried to offset these issues to the extent possible under limitations posed by paucity of data over this vast landscape.

The study was also limited by lack of field validation in support of the identified corridor routes. Future work would require radio/GPS collaring of dispersing animals to further understand movement dynamics towards validation of corridor routes suggested by this study. It will be worthwhile to caution that while our study has identified a few potential corridors for tigers, it cannot claim that the identified corridors would be the only routes that may be used by dispersing tigers. It is indeed possible that dispersing animals may also use other routes.

However given the results of this study and considering that fact that tigers by nature are habitat generalists [47], [69], [70], the proposed routes present themselves with high potential for serving as movement corridors for tigers. If RO3 is conserved and developed over a period of time as recommended by this study, it may provide a very likely route for tiger movement between the two protected areas. The MPFD can also take up work for the development of RO1 in addition to RO3 which would greatly maximize connectivity options between these two very important PA’s.

Although not specifically considered as part of this study, the development of the proposed corridor routes is also likely to serve as a dispersal corridor for another endangered species – the Asiatic wild dog or Dhole (Cuon alpinues) [71]. Like tigers, the Asiatic wild dog is also a habitat generalist and its preferred prey requirements are somewhat analogous to those of the tiger [72]. Karanth,Nichols,Karanth,Hines and Christensen [23] in their study of extinction patterns of large mammals in India have highlighted the need for creation of new protected areas and building interconnectivity between existing areas to ensure future continuity of species such as the wild dog whose historic ranges have been greatly diminished. The potential use of the proposed corridor by Asiatic wild dogs would however require further studies and validation.

Given the prevailing socio-economic conditions of the local population around most wildlife areas in India, we believe that the development and continuance of the proposed wildlife corridor between Kanha and Pench National parks cannot be sustained in the long run without active support and involvement of the local communities. We believe that an inclusive ecotourism programme with a focus on involving and benefitting local communities can be an important initiative in this direction. Karanth and DeFries [73] have reported an increase in tourist numbers to both Kanha and Pench with annual growth rates between 2002–2008 at 14.5% and 15.9% for Kanha and Pench respectively. Their data shows that domestic tourists form an overwhelming majority of annual visitors at both these locations. While opportunities emanating out of ecotourism are apparent from the above figures, there are numerous challenges that also need to be addressed. While Indian National Wildlife Action Plan mandates equitable sharing of ecotourism generated benefits with local communities, Karanth and DeFries [73] report that tourism related employment of local people living within 10 km of PA’s is currently less than 0.001%. Other studies have also suggested that contribution of ecotourism generated revenue to augment conservation programmes and improving lives of local people in many developing countries including India is highly inadequate [74], [75], [76]. If the opportunities arising out of ecotourism are to be utilized towards sustained conservation of the proposed corridor areas, inclusive enlistment of local communities through equitable benefit sharing will be vital [27]. Unless there is a value proposition for local communities in wildlife conservation via participation in ecotourism or by direct gainful employment in conservation programmes, it might be very difficult to get desired benefits ensuing from interconnection over vast landscapes. Some measures towards creating a sustainable ecotourism programme for the study area have been suggested by Rathore,Dubey,Shrivastava,Pathak and Patil [77].

We would also like to draw attention to the fact that all actions towards development of the proposed corridor should be seen in light of long term conservation goals. It would be unrealistic to expect regular movement of the focal species across the corridor in immediate future. However, with the availability of the corridor, currently isolated tiger populations within Kanha and Pench are likely to disperse and interbreed with the passage of time. It is therefore essential that a wildlife movement monitoring program be put in place which would be of great value in understanding actual corridor utilization and uncover unaccounted stressors in the dispersal of the target species.

### Conclusion

This study has attempted to identify suitable corridor routes between two important protected areas in central India. According to Chetkiewicz, Clair and Boyce [26], most of the corridor studies view corridors as structural connectivity between isolated patches and do not pay attention to their expected functionality. In the current study due emphasis has been given to elaborate functional usefulness of the corridor for the focal species. The GIS centric Least Cost Path approach was useful in modeling real world constraints such as road density, human settlement, railway density along with suitable habitat patches in identification of suitable corridors. The corridor links identified by this study if appropriately developed, have considerable potential in offering additional habitat and viable connectivity for dispersal for tigers. It is expected that the development of the corridor area will also benefit other species.

A formal report has been submitted to the State Government of Madhya Pradesh conveying the findings about probable corridor paths, weak links and recommendations for improvement and long term conservation of the corridor. According to the Indian national tiger census report released recently [7], conservation efforts within the protected national parks such as Kanha and Pench have resulted in increased population of tigers. The Indian National Tiger Conservation Authority (NTCA) in its revised guidelines to project tiger areas has places a high importance to conserving and developing tiger corridors [27]. Thus, the current findings and recommendations hold considerable importance for furthering protection of the species by providing connectivity and dispersal opportunities in long term. Prevention and management of forest fires, increasing water availability by installing water holes, and relocation of certain villages would help in conservation of the corridor. Involvement of local communities in developing an integrated ecotourism programme would help build a lasting relationship with local people whose help will be crucial in achieving conservation goals for this important wildlife area.

## Supporting Information

Table S1 **Pellet density (pellet groups/plot).**
(DOCX)

Table S2 **Pellet group density/ha.**
(DOCX)

Table S3 **Ranking of habitats on the basis of pellet density.**
(DOCX)

Table S4 **Zero counts in plots for pellet groups.**
(DOCX)

Table S5 **Segment lengths for RO1 Segments.**
(DOCX)

Table S6 **Compartments proposed for water holes.**
(DOCX)

Table S7 **Ranges and Compartments through which RO3 segments pass.**
(DOCX)
