# Supplementary material for: Opportunities of Habitat Connectivity for Tiger (Panthera tigris) between Kanha and Pench National Parks in Madhya Pradesh, India
Source: PLoS One. 2012 Jul 16;7(7):e39996. doi: 10.1371/journal.pone.0039996 (PMC3398000; doi:10.1371/journal.pone.0039996)
Supplement: Table S1 — Pellet density (pellet groups/plot). (DOCX) [file pone.0039996.s001.docx]

Table S1. Pellet density (pellet groups/plot)

| **SNo.** | **Habitat  type** | **N  (Number of plots)** | **Sambar** | **Chital** | **Wild pig** | **Gaur** | **Chowshinga** | **Nilgai** | **Barking deer** |
| --- | --- | --- | --- | --- | --- | --- | --- | --- | --- |
| 1 | BM | 102 | 0.36±0.073 | 0.69±0.135 | 0.075±0.039 | 0.13±0.042 | 0.15±0.049 | 0.15**±**0.049 | 0.04**±**0.025 |
| 2 | MB | 62 | 0.11±0.052 | 0.21±0.106 | 0.016±0.016 | 0.02±0.016 | 0.24±0.088 | 0.08**±**0.042 | 0.02**±**0.016 |
| 3 | MISC | 131 | 0.16±0.048 | 0.29±0.060 | 0.023±0.013 | 0.00±0.00 | 0.16±0.054 | 0.027 | 0.01**±**0.008 |
| 4 | TEAK | 31 | 0.32±0.156 | 0.65±0.256 | 0.032±0.032 | 0.00±0.000 | 0.06±0.045 | 0.00**±**0.000 | 0.00**±**0.000 |
| 5 | TM | 69 | 0.26±0.100 | 0.39±0.088 | 0.072±0.048 | 0.00±0.00 | 0.07±0.031 | 0.01**±**0.015 | 0.03**±**0.020 |
