# Supplementary material for: Opportunities of Habitat Connectivity for Tiger (Panthera tigris) between Kanha and Pench National Parks in Madhya Pradesh, India
Source: PLoS One. 2012 Jul 16;7(7):e39996. doi: 10.1371/journal.pone.0039996 (PMC3398000; doi:10.1371/journal.pone.0039996)
Supplement: Table S2 — Pellet group density/ha. (DOCX) [file pone.0039996.s002.docx]

Table S2. Pellet group density/ha

| **Sl. No.** | **Habitat type** | **Sambar** | **Chital** | **Wild boar** | **Bison** | **Chowshinga** | **Nilgai** | **Barking Deer** |
| --- | --- | --- | --- | --- | --- | --- | --- | --- |
| 1 | BM | 179 | 82 | 37 | 67 | 75 | 75 | 22 |
| 2 | MB | 56 | 105 | 8 | 57 | 121 | 40 | 8 |
| 3 | MISC | 80 | 145 | 11 | 0 | 80 | 27 | 4 |
| 4 | TEAK | 161 | 323 | 16 | 0 | 32 | 0 | 0 |
| 5 | TM | 130 | 196 | 36 | 0 | 36 | 7 | 14 |
