# Supplementary material for: Opportunities of Habitat Connectivity for Tiger (Panthera tigris) between Kanha and Pench National Parks in Madhya Pradesh, India
Source: PLoS One. 2012 Jul 16;7(7):e39996. doi: 10.1371/journal.pone.0039996 (PMC3398000; doi:10.1371/journal.pone.0039996)
Supplement: Table S3 — Ranking of habitats on the basis of pellet density. (DOCX) [file pone.0039996.s003.docx]

Table S3. Ranking of habitats on the basis of pellet density

| **SNo.** | **Habitat** | **Sambar** | **Chital** | **Wild Boar** | **Bison** | **Chowshinga** | **Nilgai** | **Barking Deer** | **Prey Total** |
| --- | --- | --- | --- | --- | --- | --- | --- | --- | --- |
| 1 | BM | 1 | 6 | 1 | 1 | 3 | 1 | 1 | **14** |
| 2 | MB | 6 | 5 | 6 | 3 | 1 | 2 | 3 | **26** |
| 3 | MISC | 4 | 4 | 5 | 6 | 2 | 3 | 4 | **28** |
| 4 | TEAK | 2 | 2 | 3 | 6 | 5 | 6 | 6 | **30** |
| 5 | TM | 3 | 3 | 2 | 6 | 6 | 4 | 2 | **26** |
