# Supplementary material for: Opportunities of Habitat Connectivity for Tiger (Panthera tigris) between Kanha and Pench National Parks in Madhya Pradesh, India
Source: PLoS One. 2012 Jul 16;7(7):e39996. doi: 10.1371/journal.pone.0039996 (PMC3398000; doi:10.1371/journal.pone.0039996)
Supplement: Table S4 — Zero counts in plots for pellet groups. (DOCX) [file pone.0039996.s004.docx]

Table S4. Zero counts in plots for pellet groups

| **Habitat** | **N** | **Sambar** | **Chital** | **Wild boar** | **Bison** | **Chowshinga** | **Nilgai** | **Barking Deer** |
| --- | --- | --- | --- | --- | --- | --- | --- | --- |
| BM | 102 | 47 | 60 | 63 | 58 | 58 | 58 | 64 |
| MB | 62 | 57 | 55 | 61 | 61 | 53 | 58 | 61 |
| MISC | 131 | 117 | 108 | 128 | 131 | 116 | 126 | 130 |
| TB | 35 | 32 | 17 | 34 | 32 | 31 | 35 | 35 |
| TEAK | 31 | 27 | 23 | 30 | 31 | 29 | 31 | 31 |
| TM | 69 | 61 | 50 | 66 | 69 | 64 | 68 | 67 |
| **Total** | **395** | **341** | **313** | **382** | **382** | **351** | **376** | **388** |
|  | **%** | **86** | **79** | **97** | **97** | **89** | **95** | **98** |
