# Supplementary material for: Opportunities of Habitat Connectivity for Tiger (Panthera tigris) between Kanha and Pench National Parks in Madhya Pradesh, India
Source: PLoS One. 2012 Jul 16;7(7):e39996. doi: 10.1371/journal.pone.0039996 (PMC3398000; doi:10.1371/journal.pone.0039996)
Supplement: Table S5 — Segment lengths for RO1 Segments. (DOCX) [file pone.0039996.s005.docx]

Table S5. Segment lengths for RO1 Segments

| **Segment** | **Length (Km)** |
| --- | --- |
| Segment 2 | 170 |
| Segment 3 | 21 |
| Segment 4 | 0.5 |
| Segment 5 | 16 |
