# Supplementary material for: Opportunities of Habitat Connectivity for Tiger (Panthera tigris) between Kanha and Pench National Parks in Madhya Pradesh, India
Source: PLoS One. 2012 Jul 16;7(7):e39996. doi: 10.1371/journal.pone.0039996 (PMC3398000; doi:10.1371/journal.pone.0039996)
Supplement: Table S6 — Compartments proposed for water holes. (DOCX) [file pone.0039996.s006.docx]

Table S6: Compartments proposed for water holes

| **Range** | **Compartment Cluster**  **for Proposed Water Hole** |
| --- | --- |
| Ari | Hole 1 : 162,163,164,167 |
|  | Hole 2 : 172,177,178,181 |
| Kurai | Hole 3 : 227,228,229 |
|  | Hole 4 : 235,236,252 |
|  | Hole 5 : 241,241,249 |
|  | Hole 6 : 244,245,246 |
| CL-Warraseoni | Hole 7 : 487,488,P762,P759 |
|  | Hole 8 : 474,477,478 |
| Lalburra | Hole 8 : 412,416,418,417A  Hole 9 : 405A,406A,p750,408  Hole 10 : 392,399 or (316,317,318 at BP) |
|  | Hole 11 : 698,699,700,703,704 |
|  | Hole 12 : 392,394,395,397 |
| Barghat | Hole 13 : 104,107,108,109 |
| Barghat Project  (BP) | Hole 14 : 371,377,378,379 |
|  | Hole 15 : 713,702,P23 |
|  | Hole 16 : 113,116,127,128 |
|  | Hole 1 7: 401,402,403 |
| Keolari | Hole 18 : 491,495,496,500,501 |
|  | Hole 19 : 510,516,517 |
|  | Hole 20 : 488,489,526 |
| South Lamta | Hole 21 : 1323,1324,1325 |
|  | Hole 22 : 1313,1314,1320 |
|  | Hole 23 : 1308,1309,1311 |
| North Lamta | Hole 24 : 1293,1250,1251 |
|  | Hole 25 : 1235,1233,1244 |
|  | Hole 26 : 1220,1221,1299 |
|  | Hole 27 : 1195,1197,1204 |
|  | Hole 28 : 1190,1191,1198 |
| East-Baihar | Hole 29 : 1570,1571,1574 |
|  | Hole 30 : 1566,1577 |
|  | Hole 31 : 1575,1576,1589 |
