# Supplementary material for: Opportunities of Habitat Connectivity for Tiger (Panthera tigris) between Kanha and Pench National Parks in Madhya Pradesh, India
Source: PLoS One. 2012 Jul 16;7(7):e39996. doi: 10.1371/journal.pone.0039996 (PMC3398000; doi:10.1371/journal.pone.0039996)
Supplement: Table S7 — Ranges and Compartments through which RO3 segments pass. (DOCX) [file pone.0039996.s007.docx]

Table S7. Ranges and Compartments through which RO3 segments pass

| **S. No.** | **Range Name** | **Compartment No.** |
| --- | --- | --- |
| 1 | Kurai | 223,227,228,229,232,234,235,236,237,238,240,241,242,243,244,245,246,248 |
| 2 | Ari | 165,166,168,177,181 |
| 3 | Waraseoni | 478,484,485,486A,487,488 |
| 4 | Lalbarra | 410,405A, 408, 409A, 410,411, 418, 419B,428,427,448,452,398,395,396,393A |
| 5 | Barghat Project | 700,703,713, P23, 323,325,316 |
| 6 | Keolari | 400, 483, 485, 484, 490, 492, 500,501, 502, 507, 401, P450, 512, 523 |
| 7 | Bamhani | NA |
| 8 | South Lamta | 1323,1324,1325,1327,1321,1295,1309,1308,1299 |
| 9 | North Lamta | 1263,1264,1268 |
| 10 | West Baihar | 1466,1468,1469,1463 |
| 11 | East Baihar | 1570,1569,1567,1566,1565,1575,1589,1590,1592 |
